# Association between spinal disorders and different domains of physical activity among young adult men

**DOI:** 10.3389/fspor.2022.895008

**Published:** 2022-09-08

**Authors:** Tuomas Honkanen, Jani P. Vaara, Harri Pihlajamäki, Ville Västilä, Heikki Kyröläinen

**Affiliations:** ^1^Centre for Military Medicine, Helsinki, Finland; ^2^The Department of Leadership and Military Pedagogy, National Defence University, Helsinki, Finland; ^3^Department of Orthopaedics and Traumatology, Seinäjoki Central Hospital, Seinäjoki, Finland; ^4^Department of Biology of Physical Activity, University of Jyväskylä, Jyväskylä, Finland

**Keywords:** physical activity, low back pain, neck pain, commuting physical activity, occupational physical activity, young men

## Abstract

**Background:**

There is limited evidence of how physical activity (PA) associates with low back pain (LBP) and neck pain (NP). Particularly, the association between occupational or commuting PA and LBP/NP is unclear. The aim of the present cross-sectional study was to investigate the association between spinal disorders and different physical activity domains in young and healthy adult men.

**Methods:**

Self-reported leisure time, occupational and commuting PA, as well as LBP and NP, were studied using questionnaires among young Finnish males (*n* = 1,630). Logistic regression analysis was used to study the associations of PA domains, physical fitness, and spinal disorders. Regression models were adjusted for age, education, smoking, waist circumference, and the other PA domains.

**Results:**

There was a positive association between moderate leisure-time PA and both LBP (OR: 1.51, 95% CI: 1.18–1.95) and NP (OR: 1.29, 95% CI: 1.00–1.66) compared to low PA. Furthermore, moderate (OR: 1.31, 95% CI: 1.00–1.71) and high (OR: 1.53, 95% CI: 1.15–2.02) leisure time PA groups had a higher likelihood of lumbago. Moderate (OR: 0.67, 95% CI: 0.51–0.90) and high (OR: 0.68, 95% CI: 0.48–0.95) occupational PA groups had lower likelihood for radiating LBP, while high occupational PA (OR: 0.72, 95% CI: 0.52–0.99) had lower likelihood for lumbago.

**Conclusions:**

The associations between physical activity and LBP or NP seem to vary between different domains of physical activity among young healthy men. Commuting and occupation-related PA appear not to be harmfully associated with LPB or NP, whereas moderate-level leisure time PA may be associated with increased LBP and NP, and the respective high level may be associated with an increase in the prevalence of lumbago.

## Background

Spinal disorders, particularly low back pain (LBP) (Walker, 2000) and neck pain (NP) (Croft et al., 2001) are common health problems throughout Western society. Depending on the survey, the LBP prevalence during the past 12 months ranges between 25 and 40% (Shiri and Falah-Hassani, 2017), whereas the lifetime prevalence is reported to be as high as 84% among the general population (Walker, 2000). Moreover, nearly 70% of people in the adult population will experience neck pain at some point during their lifetime (Croft et al., 2001; Covic et al., 2007). Both LBP and NP are common causes of work absence and reduced work productivity (Hartvigsen et al., 2018), and have high social and economic costs (Vlaeyen et al., 2018; Herman et al., 2019) in the general population. Therefore, it is important to study the factors that are associated with LBP and NP, particularly those health behaviors that are modifiable, such as physical activity (PA).

Physical activity (PA) is known to associate with musculoskeletal disorders, including NP (Øverås et al., 2020) and LBP (Shiri et al., 2016). PA is commonly categorized into three different domains: leisure-time (LTPA), commuting (CPA), and occupational (OPA) physical activities. LTPA is a purposeful, planned, and repetitive activity often with the goal of improving or maintaining physical fitness or other health benefits, whereas commuting physical activity (CPA) is typically described as moderate aerobic activity, such as walking or cycling, to work. Occupational physical activity (OPA) includes normally prolonged activity, such as walking, standing, and lifting during working hours at the work site (Caspersen et al., 1985). Different from the LTPA and CPA, the activity levels of OPA are not normally chosen based on personal needs and interests. The pace of the OPA is commonly set by an external factor, such as a supervisor or a machine.

Several observational studies have investigated associations between LTPA and LBP or NP; as well, several RCT studies have investigated if LTPA could protect against LBP (Shiri et al., 2016) or NP (Øverås et al., 2020) in the general population. According to the review by Øverås et al. (2020), high LTPA is associated with LBP and NP. This result is similar to a previous meta-analysis by Shiri and Falah-Hassani (2017), which included 36 cohort studies, where they reported that LTPA was associated with a risk reduction of 11–16% for chronic LBP compared to less-active individuals. Another meta-analysis by Shiri et al. (2016) suggests that moderate to a high level of LTPA has a protective effect against the development of lumbar radicular pain. However, there are conflicting results against the latest evidence (Hildebrandt et al., 2000; Chen et al., 2009; Heneweer et al., 2011). Several studies have found that vigorous physical activities have favorable effects on NP and LBP risks, whereas others have found an increased risk of LBP in individuals performing strenuous physical activity during their leisure time (Heneweer et al., 2011).

Associations between OPA and LBP or NP have also been investigated in several observational studies. According to the review by Øverås et al. (2020), increased OPA protects from LBP and NP. However, the effect of OPA on spinal disorders seems to be conflicting as well. For example, Kwon et al. (2011) were not able to find strong evidence supporting a causal relationship between OPA and LBP in their review consisting of 99 studies. Furthermore, Øverås et al. (2020) concluded in their review that OPA might have a protective role in LPB and NP in some occupations; however, the increased sitting time at work may protect against the LBP and NP among blue-collar workers.

Among the PA domains, the association of CPA with LBP/NP has been less studied, and the effects of CPA have been studied mainly with other chronic conditions, such as cardiovascular diseases. Previously, it has been reported that CPA is associated with less musculoskeletal pain (Swain et al., 2020). However, we were not able to find a study investigating associations between CPA and LBP or NP, particularly.

Although associations between LTPA or OPA and LBP or NP have been studied extensively earlier, the different domains of PA have usually not been studied within the same subject groups. Moreover, the previous research has commonly focused rather on leisure time PA without evidence on the role of other domains of PA. The previous research in this regard has typically focused on the different limited populations, such as blue-collar workers or schoolchildren but not with a nationally representative study population targeted to young adult men. The aim of the present study was to investigate the association between spinal disorders and different physical activity domains in young and healthy adult men. We hypothesized that, regardless of the PA domain, the more active subjects would have fewer spinal disorders.

## Methods

### Subjects

The study sample (*n* = 1,630) consisted of young adult Finnish men who were called up to the Finnish Defense Force's military refresher training. The present study is part of the larger reservist studies conducted in 2008 and 2015. The first group (*n* = 920) was called up to training in 2008 and the latter one in 2015 (*n* = 799). The persons called up for refresher training were different in 2008 and 2015. Participation in the study was voluntary. In 2008, out of 920 participants, 846 participated in the study, and, respectively, 784 of 799 participated in 2015. Only male subjects were included in the study because of the very small number of women in the refresher training (*n* = 0 in 2008; *n* = 15 in 2015). Furthermore, all the subjects older than 35 years were excluded from the study (*n* = 76 in 2008; *n* = 85 in 2015). The study sample has been compared with corresponding cohorts of 20- to 30-year-old Finnish men in the national register data (statistic Finland) from 2007 to 2008 for the following variables: age, education, and place of residence. The present study sample was well representative, with the exception that the Northern part of Finland was under-represented, and individuals with 13 to 15 years of education were over-presented. The background characteristics are shown in Table 1.

**Table 1:** Mean (± SD) results of background, anthropometric, and physical activity and fitness variables for each study sample alone and combined together.

	**2008 Study sample**	**2015 Study sample**	**Total study sample (2008 + 2015)**
**Background characteristics**			
Age (yrs.)	25.1 ± 4.6	26.4 ± 6.8 ^***^	25.7 ± 5.8
Smoking (%)	38.3	26.1 ^***^	32.5
Snuff use (%)	5	16.4 ^***^	10.4
*Education (yrs.)*			
≤ 9 years	2.8	3.3	3.0
10–12 years	34.6	34.0	34.3
≥ 13 years	62.5	62.8	62.6
**Anthropometric variables**			
Height (cm)	180.1 ± 6.3	179.5 ± 6.3 ^*^	179.8 ± 6.3
Body mass (kg)	80.6 ± 13.4	81.3 ± 14.9	80.9 ± 14.1
Waist circumference (cm)	86.1 ± 10.4	87.4 ± 11.1 ^*^	86.8 ± 10.7
Body mass index	24.8 ± 3.8	25.2 ± 4.1	25.0 ± 4.0
%body fat	17.9 ± 7.2	17.7 ± 7.9	17.9 ± 7.6
**Physical activity**			
**Leisure-time physical activity**			
High (%)	29.9	40.3 ^***^	34.8
Moderate (%)	38.6	29.8 ^***^	34.4
Low (%)	31.5	29.9	30.7
**Commuting physical activity**			
High (%)	34.8	28.9 ^***^	32.0
Moderate (%)	53.7	48.0 ^*^	51.0
Low (%)	11.6	23.1 ^***^	17.0
**Occupational physical activity**			
High (%)	20.9	26.1 ^*^	23.4
Moderate (%)	51.3	46.1	48.8
Low (%)	27.8	27.8	27.8
**Physical fitness**			
Sit-ups (reps/min)	38 ± 10	35 ± 12 ^***^	37 ± 11
Push-ups (reps/min)	29 ± 13	28 ± 14	29 ± 13
Maximal isometric force in bench press (N)	900 ± 199	871 ± 216 ^***^	886 ± 208
Maximal isometric force in leg press (N)	2939 ± 872	3394 ± 933 ^***^	3157 ± 930
VO_2max_ (ml·kg^−1^·min^−1^)	41.6 ± 8.1	41.1 ± 7.8	41.4 ± 8.0

*** *p*-value <0.001,

* *p*-value <0.05.

The call-up letter to military refresher training was sent to the participants 5 months prior to the training. The study plan was informed in the letter, and the protocol was further explained in detail to all the participants. The study was approved by the ethical committees of the University of Jyväskylä and the Central Finland Health Care District, as well as the Defence Command of the Finnish Defence Forces (AM5527). Written informed consent was obtained from all subjects.

### Physical activity domains

*Leisure time physical activity* (LTPA) was determined from responses to a single question: “Which of the following definitions best describe your leisure time physical activity habits? (Think of the last 3 months and consider all leisure-time physical activity that lasted at least 20 min per session”). The question had six response categories: 1 = less than once a week, 2 = no vigorous activities, but light or moderate physical activity at least once a week, 3 = brisk physical activity once a week, 4 = vigorous activity twice a week, 5 = vigorous activity three times a week and 6 = vigorous activity at least four times a week. For further analysis, the LTPA groups were categorized into 3 subgroups: Low (responses 1 and 2), Moderate (responses 3 and 4), and High (responses 5 and 6) similar to previous studies (Vaara et al., 2014; Appelqvist-Schmidlechner et al., 2020). The question about LTPA has been used previously (Fogelholm et al., 2006), and it has been validated against fitness, observing that vigorous PA showed a consistent dose-response relationship with cardiorespiratory and muscular fitness (Fogelholm et al., 2006).

*Occupational physical activity* (OPA) was determined from responses to a single question: “Which description most accurately describes your pattern of physical activity at work?” The response alternatives were 1 = mainly sitting/office work (inactive), 2 = walking around, but no lifting or carrying heavy items (low), 3 = walking around a lot, lifting and carrying heavy items, walking upstairs (moderate) and 4 = heavy physical work (high). For further analysis, the OPA groups were categorized into 3 subgroups: Low (response 1), Moderate (responses 2 and 3), and High (response 4). Good repeatability of single-item OPA questions similar to the one used in the present study has been shown in previous studies (Evenson and McGinn, 2005; Kurtze et al., 2007).

*Commuting physical activity* (CPA) was determined from responses to a single question: “How much time do you spend daily on walking or cycling from home to work or other places on a regular basis?” The response alternatives were 1 = no daily commuting physical activity (walking or cycling), 2 = one to 29-min walking or cycling daily and 3 = ≥ 30-min walking or cycling daily. According to a previous study of Appelqvist-Schmidlechner et al. (2020), this single question has not been validated; however, it has widely been used in earlier population-based studies (e.g., Barengo et al., 2006; Appelqvist-Schmidlechner et al., 2020).

### Pain and disability questionnaire

Spinal disorders for the lumbar and neck (cervical) regions were assessed by a questionnaire. The questionnaire was administered on paper at the beginning of the training. A visual analog scale was utilized (100 mm) for low back and neck pain during the last 7 days. The 0 on the scale was categorized as no pain, and 100 was the maximum imaginable pain. Furthermore, lumbago and radicular pain was determined from responses to questions: “How often have you had a lumbar radicular pain in the last month?” and “How often you have had lumbago pain in the last month?” The response alternatives were: a) none, b) 1–7 days, c) 8–14 days, d) more than 14 days but less than daily, and e) daily. The lumbago was considered here as non-radiating pain in the lumbar region with a sudden onset. All the original questions in Finnish can be found in the public report of Vaara et al. (2009).

### Statistical analysis

Means with standard deviations (± SD) were given as descriptive statistics in both study samples (years 2008 and 2015). A chi-square and a one-way repeated measured ANOVA were conducted to evaluate the difference between the samples. The data from the years 2008 and 2015 were pooled, and, thereafter, logistic regression analysis was assessed to study the associations between the low, moderate, and high categories in each PA domain and spinal disorders. Regression models were adjusted for age, education, smoking, waist circumference, and the other two PA domains. The level of significance was set at.05. All analyses were conducted using SPSS Statistics for Windows V.24.0 software.

## Results

The participants in the latter study sample of 2015 were slightly older and shorter and had larger waist circumference than their counterparts in the first study sample of 2008 (*p* < 0.05) (Table 1). In addition, there were fewer smokers but more snuff users in the study sample of 2015 compared to the study sample of 2008 (*p* < 0.05). Moreover, there were more engaged with high LTPA and less engaged with high commuting physical activity in 2015 compared to 2008 (*p* < 0.05). Furthermore, there were more involved in high occupational physical activity in 2015 compared to 2008 (*p* < 0.05). There were no differences in maximal oxygen uptake and push-ups; however, the performance in sit-ups and maximal strength tests were better in the 2008 study sample compared to 2015 (*p* < 0.001).

The prevalence of spinal disorders is presented in Table 2. There were no differences in LBP or NP or lumbar-radiating pain between the study samples. There were fewer reporting lumbago in the 2015 study sample compared to 2008 (*p* < 0.05).

**Table 2:** Mean (± SD) self-reported spinal complaints for each study sample alone and their combination.

	**2008 sample**	**2015 sample**	**Combined sample (2008 + 2015)**
	**% (n)**	**% (n)**	**% (n)**
**Self-reported complaints during the last 7 days**			
Low back pain	57.4 (823)	56.2 (733)	56.8 (1556)
Neck pain	49.6 (811)	46.3 (712)	48.1 (1523)
**Self-reported complaints during the last month**			
Lumbar radicular pain	26.1 (844)	22.3 (762)	24.3 (1606)
Lumbago	35.9 (844)	30.6 (762) ^*^	33.4 (1606)

^***^p-value < 0.001,

* *p*-value < 0.05.

Table 3 presents the associations of low, moderate, and high subgroups within each physical activity domain with LBP and NP. The moderate LTPA group had a higher likelihood of LBP and NP compared to the low LTPA group (*p* < 0.05). There were no statistically significant associations in any of the self-reported pain prevalence between the CPA or OPA groups in the fully adjusted model (Table 4). Furthermore, the moderate and high LTPA groups had a higher likelihood of lumbago compared to the low LTPA group (*p* < 0.05). Whereas, the moderate and high OPA groups had a lower likelihood of radiating compared to the low LTPA group (*p* < 0.05), while the high OPA had a lower likelihood of lumbago compared to the low OPA group (*p* < 0.05).

**Table 3:** The associations between low, moderate, and high subgroups according to each physical activity domain and self-reported low back pain and neck pain adjusted for age, education, smoking, waist circumference, and the other two physical activity domains.

**Physical activity**			**Low back pain during last 7 days**		**Neck pain during last 7 days**
	**Subgroup**	**n**	**Odds Ratio (95 CI)**	**n**	**Odds Ratio (95 CI)**
**LTPA**	Low	518	1.0 Ref.	502	1.0 Ref.
	Moderate	516	1.51 (1.18–1.95)^**^	503	**1.29 (1.00–1.66)** ^*^
	High	447	1.19 (0.91–1.56)	444	1.25 (0.95–1.64)
**CPA**	Low	475	1.0 Ref.	462	1.0 Ref.
	Moderate	496	1.04 (0.81–1.35)	488	1.16 (0.89–1.66)
	High	510	1.12 (0.87–1.46)	499	1.19 (0.92–1.54)
**OPA**	Low	342	1.0 Ref.	335	1.0 Ref.
	Moderate	727	0.80 (0.62–1.04)	711	0.78 (0.60–1.02)
	High	412	1.00 (0.73–1.35)	403	1.22 (0.90–1.66)

* *p*-value <0.05,

** *p*-value < 0.005,

^***^p-value < 0.001.

**Table 4:** The associations between low, moderate, and high subgroups according to each physical activity domain and self-reported lumbar radicular pain and lumbago complaints adjusted for age, education, smoking, waist circumference, and physical activity domains.

**Physical activity**			**Lumbar radicular pain during the last month**		**Lumbago during the last month**
	**Subgroup**	***n***	**Odds Ratio (95 CI)**	***n***	**Odds Ratio (95 CI)**
**LTPA**	Low	532	1.0 Ref.	532	1.0 Ref.
	Moderate	536	0.83 (0.62–1.12)	536	**1.31 (1.00–1.71)** ^*^
	High	467	0.85 (0.63–1.15)	467	**1.53 (1.15–2.02)** ^*^
**CPA**	Low	491	1.0 Ref.	491	1.0 Ref.
	Moderate	510	0.98 (0.73–1.32)	510	1.24 (0.95–1.63)
	High	534	0.93 (0.69–1.25)	534	1.28 (0.98–1.68)
**OPA**	Low	357	1.0 Ref.	357	1.0 Ref.
	Moderate	754	**0.67 (0.51–0.90)** ^*^	754	0.92 (0.70–1.20)
	High	424	**0.68 (0.48–0.95)** ^*^	424	**0.72 (0.52–0.99)** ^*^

* *p-*value < 0.05,

^**^p-value < 0.005,

^***^p-value < 0.001.

All the results were adjusted for age, education, smoking, waist circumference, and the other two PA domains.

## Discussion

The main findings of the present study demonstrate that individuals with moderate LTPA had a higher likelihood of LBP and NP compared to low LTPA. Furthermore, moderate as well as high LTPA had a higher likelihood of lumbago compared to the low LTPA group. Moreover, moderate and high OPA had a higher likelihood of radiating LBP, while high OPA had a lower likelihood of lumbago compared to the low OPA groups. The LBP or NP did not associate with CPA in any PA subgroups (Low, Moderate, or High).

The present findings, concerning the association between LTPA and spinal disorders, were partly conflicting compared to previous studies (Shiri et al., 2016; Shiri and Falah-Hassani, 2017; Øverås et al., 2020). For example, Shiri and Falah-Hassani (2017) found in their review of 36 prospective cohort studies that high LTPA was not associated with LBP in the past month. This is partly in line with our findings where only moderate, not high-level LTPA, was not associated with LBP. Moreover, Øverås et al. (2020) found in their review that increased LTPA would not protect from LBP or NP; instead, it may even slightly increase the risk. Similarly, we were able to conclude that moderate and high LTPA associated positively with lumbago. In contrast to our results, some reviews have found that moderate or high LTPA is associated with a reduced risk of LBP (Alzahrani et al., 2019), or NP (Kim et al., 2018). LTPA has also been suggested to have a protective effect against the development of lumbar radicular pain (Shiri et al., 2016), and, furthermore, reduce the risk of chronic LBP (Shiri and Falah-Hassani, 2017), which is not in line with the present results. The reason why the moderate LTPA group had significantly higher odds of LBP compared to the low LTPA group is not clear. However, it may be due to the fact that the non-chronic LBP episode is a common condition in the general population, and, therefore, this type of outcome can be a part of a normal healthy lifestyle and not related to physical activity to a large extent.

It has been reported in previous cross-sectional studies that high OPA (Bjorck-van Dijken et al., 2008) and overall PA (Heneweer et al., 2009) associate with an increased LBP among the general population. Furthermore, there is evidence that increased sitting time at work reduces the risk of NP and LBP among blue-collar workers (Øverås et al., 2020). This is against our findings that lumbar radicular pain and lumbago experienced during the last month are negatively associated with high OPA in our present study in young healthy adult men. Moreover, we found that high OPA is not associated with LBP or NP. This may be due to different and limited subject groups (i.e., industrial workers, health care workers, etc.) in previous studies compared to our sample of young healthy men.

There was no association between any CPA and LBP (overall pain, lumbago, or radiating) or NP in the present study. As stated earlier, CPA has been found to associate with less musculoskeletal pain (Swain et al., 2020). However, this report investigates the relationship between transportation and overall pain, not particularly LBP or NP. Although the association between CPA and other chronic conditions is studied extensively, we were not able to find a study investigating the association between CPA and LBP or NP. The reason for the lack of association between CPA and spinal disorders may be due to the low amount and intensity of CPA in the high subgroup. The high subgroup of the CPA subgroup included all the subjects with total walking and/or cycling > 29 min daily, which may be considered a relatively small amount of activity when combining all daily commuting. Furthermore, the intensity level or amount (km walked/bicycled) was not defined. The total > 29 min may consist of only a low-level physical activity such as walking.

The comparison between the results of the present and previous cross-sectional studies has to be done with care. Previous studies have classified the levels of LTPA in different ways and used different cut points for moderate and high levels of LTPA (Shiri and Falah-Hassani, 2017). In addition, the intensity and the type of LTPA may vary between and within the studies where the LTPA is. It is clear that the exposure misclassification can lead to under or overestimation of an association between LTPA and LBP, and, thereafter, conflicting results between studies. Moreover, the cause of LBP is multifactorial, and its etiology is uncertain. There are meta-analyses investigating the relationship between LBP and specific occupational activities. For example, the relationship between LBP and occupational lifting (Wai et al., 2010a) or bending/twisting (Wai et al., 2010b) has been studied widely. Although there is moderate evidence of an association between LBP and specific types of lifting activities, it is unlikely that occupational lifting is independently causative of LBP (Wai et al., 2010a). Moreover, there is no evidence for causation for either occupational bending or twisting and LBP (Wai et al., 2010b).

Moreover, when comparing the present and previous results, it has to be kept in mind that the association between PA and spinal disorders has been previously investigated in substantially varying groups of subjects. For example, previous studies have included blue-collar workers (Hallman et al., 2016, 2017; Korshøj et al., 2018), schoolchildren (Kujala et al., 1999; Auvinen et al., 2007), and patients with LBP (Hendrick et al., 2013), but not healthy young adult men as their subjects. This has an affect on conflicting results because individuals with a history of either frequent or chronic LBP are more likely to become less active during their leisure time than those without LBP (Leeuw et al., 2007). Moreover, Shiri and Falah-Hassani (2017) report in their meta-analysis that most of the previous studies have not been able to control any confounding factors (age, education, smoking, waist circumference, and the other two PA domains).

The present study provides novel results of associations between different domains of PA and LBP or NP. Although the relationship between independent associations of different (LTPA, OPA, and CPA) PA and other chronic conditions (i.e., cardiovascular diseases) has been studied extensively, the association between three different domains and LBP or NP has not been studied previously. Recent studies have been restricted to either LTPA or OPA individually (Øverås et al., 2020). For example, Øverås et al. (2020) were able to include only one article (Hallman et al., 2016) in their review reporting on the independent effects of two different PA domains (LTPA and OPA) within the same study sample.

The mechanisms underlying the protective effect of LTPA on LBP or NP are not clear. The preventive mechanisms are commonly proposed to work by changing muscle activation and/or posture. It is also logical to assume that more muscle strength would give more support and protect the vulnerable structures (i.e., intervertebral discs), especially in strenuous activities. In contrast, several reviews assess the aspects of physical activity at work as a possible risk factor in LBP. The biomechanical loading at work (such as heavy lifting and bending and twisting) is proposed to cause spinal disorders. Nowadays, spinal disorders are also understood from a biopsychosocial perspective, involving combinations of psychological, social, lifestyle, and physical factors (Kamper et al., 2015). However, the previous physical activity investigations for spinal disorders (including our present study) have not measured biopsychosocial variables. Therefore, future research should involve measuring an array of biopsychosocial variables to further our understanding of the mechanisms between spinal disorders and PA.

The strength of the present study is the extensive data set where the associations between each PA domain and self-reported LBP and NP were well adjusted for several confounding factors (age, education, smoking, waist circumference, and the other two physical activity domains). However, there are some limitations to be considered. The level of PA and prevalence of disorders were both based on subjective measurements (questionnaires). It has been reported that subjective measurements may lead to under- or overestimation of PA, which may result in bias in the association between PA and spinal disorders (Verbunt et al., 2009). An objective measurement conducted with activity monitors would have included several advantages such as having greater validity and providing both qualitative and quantitative analysis of PA with minimal burden on participants. However, previous studies conducted with objective measures, such as accelerometers, have had limited registration time compared to questionnaires. Finally, as a cross-sectional study, no suggestions for causal effects or direction of causality between PA and LBP or NP can be made.

## Conclusions

The associations between physical activity and LBP or NP seem to vary between different domains of physical activity. CPA and OPA appear not to be harmfully associated with LPB or NP. Whereas moderate-level LTPA may increase the odds of having LBP and NP, as well as high level, may increase the prevalence of lumbago. In conclusion, LTPA showed more contentious findings in LTPA behavior regarding both LBP and NP pain, and, therefore, CPA and OPA could be recommended, while LTPA requires more focus on its safety and a correct performance technique to minimize the risk of LBP and NP.

## Data availability statement

The raw data supporting the conclusions of this article will be made available by the authors, without undue reservation.

## Ethics statement

The studies involving human participants were reviewed and approved by the Ethical Committees of the University of Jyväskylä and the Central Finland Health Care District, as well as the Defence Command of the Finnish Defence Forces (AM5527). The patients/participants provided their written informed consent to participate in this study.

## Author contributions

TH and JV took part in all the elements of the study and drafted the manuscript. VV took part in data collection. HP and HK contributed to the design of the study, data interpretation, and statistical analyses. All authors read and approved the final manuscript.

## Conflict of interest

The authors declare that the research was conducted in the absence of any commercial or financial relationships that could be construed as a potential conflict of interest.

## Publisher's note

All claims expressed in this article are solely those of the authors and do not necessarily represent those of their affiliated organizations, or those of the publisher, the editors and the reviewers. Any product that may be evaluated in this article, or claim that may be made by its manufacturer, is not guaranteed or endorsed by the publisher.

## Abbreviations

PA: physical activity
LTPA: leisure time physical activity
CPA: commuting physical activity
OPA: occupational physical activity
LBP: low back pain
NP: neck pain.
